# Comparison of the Efficacy of a Real-Time and Offline Associative Brain-Computer-Interface

**DOI:** 10.3389/fnins.2018.00455

**Published:** 2018-07-11

**Authors:** Natalie Mrachacz-Kersting, Susan Aliakbaryhosseinabadi

**Affiliations:** Center for Sensory-Motor Interaction, Department of Health Science and Technology, Faculty of Medicine, Aalborg University, Aalborg, Denmark

**Keywords:** human, plasticity, brain-computer-interface, offline, online, Hebbian plasticity, tibialis anterior

## Abstract

An associative brain-computer-interface (BCI) that correlates in time a peripherally generated afferent volley with the peak negativity (PN) of the movement related cortical potential (MRCP) induces plastic changes in the human motor cortex. However, in this associative BCI the movement timed to a cue is not detected in real time. Thus, possible changes in reaction time caused by factors such as attention shifts or fatigue will lead to a decreased accuracy, less pairings, and likely reduced plasticity. The aim of the current study was to compare the effectiveness of this associative BCI intervention on plasticity induction when the MRCP PN time is pre-determined from a training data set (BCI_offline_), or detected online (BCI_online_). Ten healthy participants completed both interventions in randomized order. The average detection accuracy for the BCI_online_ intervention was 71 ± 3% with 2.8 ± 0.7 min^-1^ false detections. For the BCI_online_ intervention the PN did not differ significantly between the training set and the actual intervention (t_9_ = 0.87, *p* = 0.41). The peak-to-peak motor evoked potentials (MEPs) were quantified prior to, immediately following, and 30 min after the cessation of each intervention. MEP results revealed a significant main effect of time, *F*_(2,18)_ = 4.46, *p* = 0.027. The mean TA MEP amplitudes were significantly larger 30 min after (277 ± 72 μV) the BCI interventions compared to pre-intervention MEPs (233 ± 64 μV) regardless of intervention type and stimulation intensity (*p* = 0.029). These results provide further strong support for the associative nature of the associative BCI but also suggest that they likely differ to the associative long-term potentiation protocol they were modeled on in the exact sites of plasticity.

## Introduction

Since Daly et al. (2009) proposed the possibility of a Brain-Computer-Interface (BCI) designed for neuromodulation of stroke patients, the field has rapidly expanded with numerous novel BCIs being introduced and tested in the clinic (Ang et al., 2010; Broetz et al., 2010; Cincotti et al., 2012; Li et al., 2013; Ramos-Murguialday et al., 2013; Mukaino et al., 2014; Young et al., 2014; Pichiorri et al., 2015; Mrachacz-Kersting et al., 2016). To date the main focus has been on upper limb rehabilitation with relatively few targeting lower limb function (for a review see, Teo and Chew, 2014; Cervera et al., 2018). In addition, only one group has investigated patients in the sub-acute phases of stroke (Mrachacz-Kersting et al., 2017b), presumably due to the relatively stable condition that a chronic stroke patient presents. Effects from the use of a BCI are thus easier to control since patients in the acute and subacute phase are prone to spontaneous biological recovery (Krakauer and Marshall, 2015).

Typically, BCIs function by collecting the brain signals during a specific state such as performing a movement or motor imagery, extracting features of interest and then translating these into commands for external device control (Daly and Wolpaw, 2008). The available non-invasive BCIs for stroke patients have implemented both electroencephalography (EEG) or near-infrared spectroscopy (NIRS) to acquire the brain signals, extracted various spectral and temporal features [e.g., sensorimotor rhythm, movement related cortical potentials (MR)] and provided diverse types of afferent feedback to the patient such as those generated from using robotic devices, virtual reality or by driving direct nerve or muscular electrical stimulation (for review see, Cervera et al., 2018).

A vital component of any BCI designed for rehabilitation of lost motor function in stroke patients, is that the physiological theories behind learning and memory must be satisfied. One of the most influential theories was proposed in 1949 by Hebb (2005) from which we know that “*Cells that fire together, wire together.*” Although Hebb proposed his theory on theoretical grounds, animal data later verified that if the pre-synaptic neuron is activated simultaneously with the post-synaptic cell, plasticity is induced, often referred to as long-term potentiation (for a review see, Cooke and Bliss, 2006). In 2000, a group from Rostock University were the first to demonstrate long-term potentiation like plasticity in the intact human brain (Stefan, 2000) with later applications to lower limb muscles (Mrachacz-Kersting et al., 2007). In this intervention [paired associative stimulation (PAS)], a peripheral nerve that innervates the target muscle is activated using a single electrical stimulus and once the generated afferent volley has arrived at the motor cortex, a single non-invasive transcranial magnetic stimulus (TMS) is provided to that area of the motor cortex that has a direct connection to the target muscle (for a review see, Suppa et al., 2017).

In a modified version of PAS, the TMS stimulus has been replaced by the movement related cortical potential (MRCP) (Mrachacz-Kersting et al., 2012). The MRCP, that can be readily measured using EEG, is a slow negative potential that arises approximately 1–2 s prior to movement execution or imagination and attains its peak negativity at the time of movement execution (Walter et al., 1964). This intervention, also termed an associative BCI, induces significant plasticity of the cortical projections to the target muscle and leads to significant functional improvements in chronic and subacute stroke patients (Mrachacz-Kersting et al., 2016, 2017b). In the first phase, patients are asked to attempt 30–50 movements (dorsiflexion of the foot), timed to a visual cue and they receive no sensory feedback. The time of the peak negativity (PN) of the resulting MRCP for every trial is extracted and an average calculated. During the second phase (the actual associative BCI intervention), this time is used to trigger the electrical stimulation of the target nerve such that the generated afferent volley arrives at the motor cortex at precisely peak negativity. Typically, 30–50 such pairings are performed over 3–12 sessions. Since the trigger of the electrical stimulator is not based on the online detection of the MRCP during the second phase, this intervention does not represent a BCI in the classical sense. In the current study the aim was to compare the effects of this associative BCI intervention on plasticity induction as quantified by the motor evoked potential (MEP) following TMS when the MRCP PN time is determined from the phase one trials (BCI_offline_ modus) or detected during the second phase by using the phase one trials as a training data set (BCI_online_ modus).

## Materials and Methods

### Participants

Ten participants (four females and six males, average age: 22.3 ± 1.2 years) without any known physical or neurological disorders all participants were classified as right side dominant with a mean laterality quotient of 0.97 (range: 0.59–1) according to the Edinburgh handedness inventory questionnaire (Oldfield, 1971). This study was carried out in accordance with the recommendations of the Scientific Ethics Committee of Northern Jutland guidelines. The protocol was approved by the Scientific Ethics Committee of Northern Jutland (Reference number: VN-20070015). All subjects gave written informed consent in accordance with the Declaration of Helsinki.

### Apparatus and Instrumentation

#### Surface Electromyography

The electromyographic (EMG) activity of the target muscle, the tibialis anterior (TA) on the dominant side was quantified using disposable surface electrodes (Neuroline 720, Ambu, Ambu A/S, Denmark) that were placed according to the SENIAM guidelines^1^. For quantification of plasticity induction using non-invasive TMS, the EMG amplifier pod supplied by Rogue Research Inc. as part of the Brainsight^TM^ system (Rogue Research, Inc.), was used to collect MEP data. During the BCI intervention, a single channel EMG was recorded to control for the participant’s movement using the g.USBamps (g.tec GmbH, Austria) at a sampling frequency of 256 Hz.

### Electroencephalography (EEG)

Monopolar EEG was obtained from 10 channels (FP1, Fz, FC1, FC2, C3, Cz, C4, CP1, CP2, and Pz according to the standard international 10–20 system) with the reference electrode on Fz and ground on the left earlobe. Channel Cz was the central channel based on the large Laplacian (McFarland et al., 1997). Signals were acquired using an active EEG electrode system (g. GAMMAcap^2^, Austria) and g.USBamp amplifier (gTec, GmbH, Austria) at a sampling frequency of 1200 Hz (16 bits accuracy) and a hardware filter of 0 to 100 Hz.

#### Electrical Stimulation (ES)

The deep branch of the common peroneal nerve (dCPN) was stimulated using disposable surface electrodes (32 mm, PALS^®^ Platinum, Patented Conductive Neurostimulation Electrodes, Axelgaard Manufacturing, Co., Ltd., United States) with the cathode proximal. A NoxiTest isolated peripheral stimulator (IES 230) supplied single pulses (1 ms width, 20–30 mA) every 3–5 s while a suitable stimulating position (where the TA M-wave attained the highest peak to peak amplitude and activity pf the synergistic peroneal muscles and the antagonist soleus was minimal) was determined. Next, the motor threshold was quantified as that stimulating intensity where an M-wave became visible in the TA EMG. This intensity was used in the subsequent BCI interventions (refer to see section “Associative BCI Interventions”).

#### Transcranial Magnetic Stimulation (TMS)

To quantify the TA MEP, single TMS pulses with a posterior to anterior directed current were applied using a Magstim 200 (Magstim Company, Dyfed, United Kingdom) and a focal figure of eight coil (110 mm diameter).

### Experimental Procedures

Participants attended two separated sessions spaced at least 48 h apart. Each session was comprised of pre-measures where TA MEP sizes were quantified, phase one and two of the associative BCI intervention, and post and 30 min post-measures of TA MEPs. During all parts, participants were seated in a comfortable chair with both feet resting on foot plates.

Following EMG electrode placements, the optimal placement of the TMS coil was determined using a stimulator output of 50%. Three stimuli were initially provided over the vertex and the peak to peak size of the TA MEP monitored online. This was repeated for 3–5 positions around the vertex and the site that resulted in the largest and most consistent TA MEPs deemed the hotspot. To ensure that the stimulation was always applied over the same area of the motor cortex the coil position was maintained by marking this spot using Brainsight^TM^ (Rogue Research, Inc.). Next the resting motor threshold (RMT) was established which was the highest stimulation intensity that produced TA MEPs with an amplitude of at least ∼50 μV while the muscle was at rest, in 5 out of 10 consecutive stimuli. Finally, 10 stimuli were provided randomly every 5–7 s at each intensity of 90, 100, 110, 120, 130, and 140% RMT (total of 60 stimuli).

**FIGURE 1:**
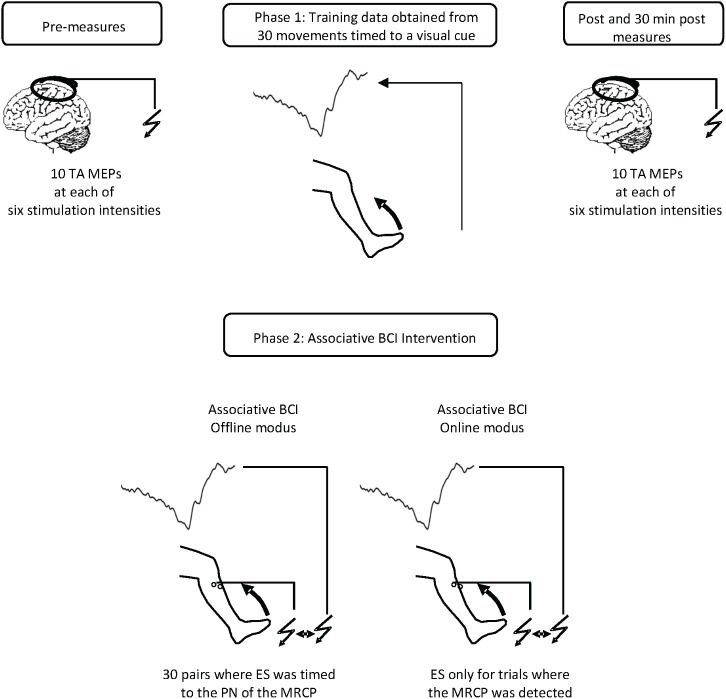
Overview of the intervention sessions. Prior to the interventions 10 TMS stimuli were applied at each of six different intensities. The interventions (spaced at least 48 h apart) consisted of two phases. In phase one participants completed 30 dorsiflexion movements while EEG data were collected. In phase two, participants were exposed to concurrent motor imagination and peripheral nerve stimulation. In the associative BCI_offline_ intervention, the stimulation was provided during each motor imagery trial and the timing set in relation to the peak negativity obtained from the EEG data of phase one. In the associative BCI_online_ intervention, the stimulation was only provided if an MRCP was detected. The detection algorithm was trained from the data obtained in phase one. For each modus participants completed 30 imagery trials. Immediately following and 30 min after the cessation of the interventions, another 10 TMS stimuli were applied at each of six different intensities.

Following the pre-measures, the participants were prepared for EEG recordings and once completed, were exposed to one of the associative BCI interventions as outlined in Section “Associative BCI Interventions.” The EEG cap was then removed, and the post and 30 min post TA MEP measures taken (i.e., 10 stimuli provided randomly every 5–7 s at each intensity of 90, 100, 110, 120, 130, and 140% RMT (total of 60 stimuli)). **Figure 1** provides an overview of the intervention sessions.

### Associative BCI Interventions

#### Phase One

Phase one of each session was the same for all participants regardless of the intervention. A cue provided on a computer screen placed at least 1.5 m in front of the participant indicated when to prepare, execute, and release a single ballistic dorsiflexion of the dominant foot. The cue consisted of five parts, (1) The word ‘Focus’ appeared (duration randomized between 2 and 3 s), (2) The drawing of a ramp appeared where the initial 2 s prior to the upwards turn served as the preparation time, (3) The upwards turning part of the ramp indicated when to execute the movement, (4) A holding phase of 2 s where the new position had to be held and 5. The word ‘Rest’ appeared (duration randomized between 4 and 5 s). A total of 30 such movements were performed.

#### Phase Two

This phase differed between the two sessions depending on whether the participant was exposed to the offline (BCI_offline_) or online (BCI_online_) modus of the associative BCI intervention as outlined below.

#### BCI_offline_ Session

The onset of each movement was quantified from the TA EMG data and the continuous EEG data divided into epochs of 4 s (2 s prior to and 2 s following the onset of the movement). A band pass filter (0.05–10 Hz) and a Laplacian channel (McFarland et al., 1997) was used to enhance the MRCP in each epoch. Next, each epoch where the PN was not within a time window of -500 to 500 ms or contained electrooculographic (EOG) activity exceeding 70 mV were discarded. For the remaining epochs, the time of PN was extracted and averaged. This time was used during phase two to time the onset of the electric stimulator. More precisely, the timing was calculated as the mean PN-50 ms. The 50 ms represents the mean latency for the afferent inflow resulting from the peripheral stimulus to reach the somatosensory cortex plus a cortical processing delay and is based on previous work (Mrachacz-Kersting et al., 2007). Following the quantification of the PN, participants were asked to complete another 30 movements as for phase one, however this time imagined, and timed to the cue as for phase one. During each repetition they also received a single electrical stimulus as outlined in Section “Electrical Stimulation (ES).” In the offline modus, phase two thus contained 30 pairings of the MRCP and ES.

#### BCI_online_ Session

The EEG signals recorded in phase 1 were filtered [2^nd^ order band-pass Butterworth filter (0.05–5 Hz)]. The EEG signals in the range of (-2 1) s with regards to movement onset were considered as ‘signal intervals’ while the remaining data were ‘noise intervals.’ Next, spectral and temporal analysis was performed on each trial of both signal and noise intervals to extract 25 spectral and 17 temporal features. This procedure was repeated for all recorded channels.

**FIGURE 2:**
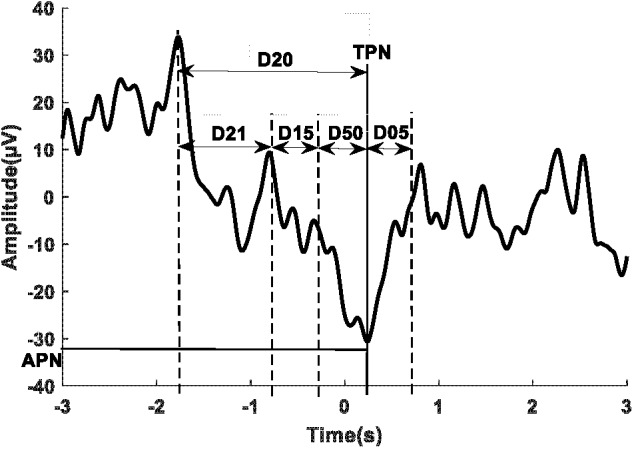
A sample of a single trial of the MRCP with the time domains used for feature extraction. D21: [–2 –1] s, D15: [–1 –0.5] s, D50: [–.5 0] s, D20: [–2 0] s, and D05: [0.5] s where 0 represents the peak negativity obtained from the onset of the movement.

Twenty-five spectral features were computed from the power of the EEG trials in five main frequency ranges; Delta (0.05–3 Hz), Theta (4–8 Hz), Alpha (8–13 Hz), Beta (1331 Hz), and Gamma (32–100 Hz). These were extracted from five time intervals; [-2 0] s, [-2 -1] s, [-1 0] s, [-1 -0.5] s, and [-0.5 0] s with respect to the movement onset obtained from EMG signals. Seventeen temporal features were obtained from each trial by extracting the time and amplitude of the peak negativity of the MRCP. Pre-movement slopes were attained from linear regression in five time intervals; [-2 -1] s, [-2 0] s, [-1 0] s, [-1 -0.5] s, and [-0.5 0] s where 0 is the time of peak negativity. In addition, the variability of the MRCP defined as the standard deviation as well as the average MRCP across all trials were computed in the same five time ranges. **Figure 2** visualizes the time intervals implemented as well as the amplitude and time of peak negativity. Lastly, 27 tempo-spectral features were extracted by combining temporal and spectral features.

These features were subsequently used as the input for three types of classifiers, K-nearest neighbor (KNN, five neighbor points with Euclidean distance), Support vector machine (SVM, 2^nd^ order polynomial as the kernel function with automatic scale) and Decision Tree (the split criteria was Gini’s diversity index). Data were classified to either signal or noise by applying fivefold cross validation divided into fivefold (4 for training and 1 for testing). The classification output for all channels was computed and the three channels with the highest accuracy and corresponding classifier and feature type was selected. In phase two of the intervention, the continuous incoming data of the selected channels (3 s long with 2.5 s overlapping) were classified by using the selected features and classifiers. The decision was made if more than one channel showed one of the two classes. True and false detections were recorded during phase two of the BCI_online_ session and used to calculate the true positive rate (TPR), false positive (FP), true negative rate (TNR), and false negative (FN) to assess BCI performance.

### Statistical Analysis

To quantify the reliability of the PN time of the MRCP as well as the number of pairings of MRCP and ES for the BCI_offline_ session, a Student’s paired *t*-test was applied. To ensure that the pre-intervention MEP values were matched between sessions, a two-way repeated analysis of variance (rmANOVA) was conducted with the factors intervention (BCI_offline_ and BCI_online_) and TMS stimulation intensity (90, 100, 110, 120, 130, and 140% RMT). A three-way rmANOVA with the factors time (pre, post and 30 min post-intervention), intervention (BCI_offline_ and BCI_online_) and TMS stimulation intensity (90, 100, 110, 120, 130, and 140% RMT), tested the effectiveness of the two interventions in inducing alterations of the corticospinal tract excitability. Greenhouse–Geisser corrections were used in the case of sphericity being violated. The significance level was set to *p* < 0.05.

## Results

### MRCP Reliability

**FIGURE 3:**
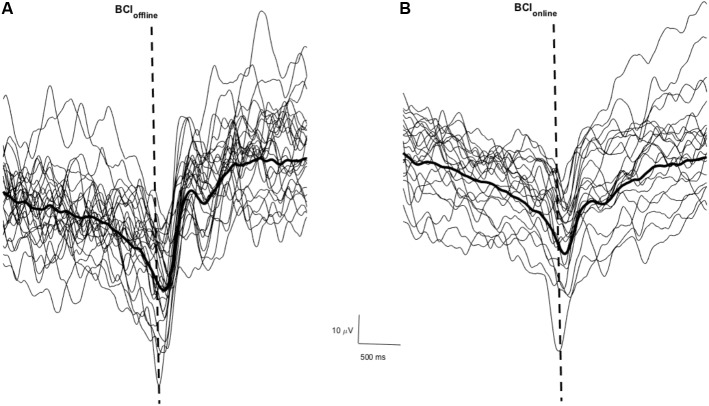
Single trial MRCPs and the average MRCP for one participant for the BCI_offline_ **(A)** and BCI_online_ **(B)** interventions respectively. The dashed vertical line indicates the time of the cue to perform the movement. Trials with EOG activity have been removed.

**Figure 3** shows a sample of the MRCP of single trials (thin traces) and the average across all trials (thick trace) for one participant during phase one of the BCI_offline_ (**Figure 3A**) and BCI_online_ (**Figure 3B**) experimental sessions respectively. The dashed vertical lines indicate the time of the cue to move. Across all participants the PN of the MRCP attained values of -10 ± 70 ms (BCI_offline_ session) and -20 ± 60 ms (BCI_online_ session). A Student’s paired *t*-test revealed no significant differences between sessions (t_9_ = 1.68, *p* = 0.13).

### BCI Performance During Phase Two of the Associative BCI_offline_ and BCI_online_ Interventions

The time of PN of the MRCP during phase two of the BCI_offline_ session was -10 ± 40 ms which was not significantly different to those values attained during phase one (t_9_ = 0.87, *p* = 0.41). **Table 1** displays TPR, FP, TNR, and FN in phases 1 and 2 of the BCI_online_ session for single participants.

**Table 1:** TPR, FP, TNR, and FN in phases 1 and 2 of the BCI_online_ session for single participants.

	Phase 1				Phase 2			
		
Participant	TPR	FP	TNR	FN	TPR	FP	TNR	FN
1	84	1.5	86	1.7	73	2.4	78	3.0
2	83	1.6	85	1.8	72	2.4	78	3.1
3	80	2.1	81	2.2	73	2.7	75	2.3
4	88	0.9	92	1.3	77	1.5	86	2.5
5	81	1.9	83	2.3	70	2.4	78	3.3
6	81	1.9	83	2.1	71	2.7	75	3.2
7	78	2.3	79	2.4	68	3.2	71	3.5
8	80	2.0	82	2.2	71	2.8	74	3.2
9	79	2.2	80	2.3	68	4.2	72	3.5
10	77	2.3	75	2.5	67	3.3	70	3.6

**Average**	81.1	1.9	82.6	2.1	71.0	2.8	75.7	3.1
***SD***	3.2	0.4	4.6	0.4	3.0	0.7	4.6	0.4


The performance of the BCI in the BCI_online_ session for all participants expressed as TPR, TNR, FP, and FN respectively, were 71 ± 3, 76 ± 5% and 2.8 ± 0.7, 3.1 ± 0.4 min^-1^.

### Changes in the Output Properties of the Motor Cortex Following the Associative BCI_offline_ and BCI_online_ Interventions

Prior to the interventions, the amplitude of the TA MEPs induced at the highest stimulation intensity across all participants were 515 ± 186 and 464 ± 164 μV (mean ± SE) for the BCI_offline_ and BCI_online_ training interventions, respectively. There was no significant interaction between intervention and stimulation intensity, *F*_(5,45)_ = 0.47, *p* = 0.799 for the pre-intervention measures. The experimental sessions started with a similar baseline excitability across all participants since the main effect of intervention was not significant, *F*_(1,9)_ = 0.048, *p* = 0.83, after pooling the interaction term.

**FIGURE 4:**
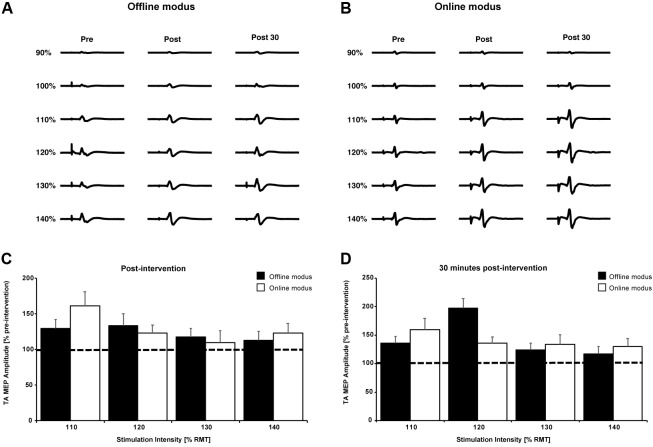
Single TA MEP traces for 90–140% RMT prior to, following, and 30 min after the BCI_offline_ **(A)** and BCI_online_ **(B)** interventions for one participant. **(C,D)** Mean TA MEP amplitudes for 110–140% RMT across all participants immediately following and 30 min after both interventions. Data are expressed as a percentage of pre-intervention values (black dashed line). Black bars represent the offline modus and the white bars represent the online modus. Error bars represent SEM.

**Figures 4A,B** show single TA MEP traces from one participant prior to, immediately following and 30 min after the cessation of the BCI_offline_ and BCI_online_ training. **Figures 4C,D** contain the mean TA MEP amplitudes across all participants following and 30 min after the BCI_offline_ and BCI_online_ training interventions for all stimulation intensities, expressed as a percentage of the pre-intervention TA MEP amplitudes for all stimulation intensities.

The three-way interaction and all two-way interactions were not significant (all *p*’s ≥ 0.26). After pooling the two- and three-way interaction terms, there was a significant main effect of time, *F*_(2,18)_ = 4.46, *p* = 0.027. The mean TA MEP amplitudes were significantly larger 30 min after (277 ± 72 μV) the BCI interventions compared to pre-intervention MEPs (233 ± 64 μV) with *p* = 0.029 (Bonferroni *post hoc* analysis). There was no significant difference between TA MEP amplitudes immediately following and pre-intervention (*p* = 0.148).

As expected, there was a significant main effect of stimulation intensity, *F*_(5,45)_ = 5.323, *p* = 0.001. The average TA MEP amplitudes were significantly larger at stimulation intensities of 140% RMT (463 ± 162 μV) compared to 130% (405 ± 135 μV), 120% (271 ± 61 μV), 110% (189 ± 27 μV), 100% (112 ± 13 μV), and 90% RMT (63 ± 10 μV) regardless of intervention type and stimulation time (all *p*’s ≤ 0.037, Bonferroni *post hoc* analysis). TA MEP amplitudes were also significantly larger at stimulation intensities of: 130% RMT compared to 120, 110, 100, and 90% RMT (all *p*’s ≤ 0.047); 120% RMT compared to 110, 100, and 90% RMT (all *p*’s ≤ 0.02); 110% RMT compared to 100 and 90% RMT (both *p*’s ≤ 0.02); and 100% compared to 90% RMT (*p* < 0.026).

MEP changes occurred independently of the type of BCI intervention used since there was no significant main effect of intervention, *F*_(1,9)_ = 0.057, *p* = 0.816. These analyses demonstrate the effectiveness of both BCI interventions in inducing significant neurophysiological changes. Both BCI interventions resulted in a significant increase of the TA MEP amplitude that outlasted the intervention time by at least 30 min.

## Discussion

The aim of the current study was to compare the effects of an associative BCI intervention on plasticity induction when the MRCP PN time is pre-determined from a training data set (BCI_offline_), or detected online (BCI_online_). The results show that both interventions resulted in significant increases in the cortical projections to the target muscle.

### BCI Performance During Phase Two of the Associative BCI_offline_ and BCI_online_ Interventions

One of the advantages of asking participants to perform the BCI task to a cue is that it facilitates motor imagery or motor attempt (Heremans et al., 2009). Hence in our previous studies, we used the initial training data set to quantify the timing of the ES. Aside from the lower computation power required, this also ensures that patients do not become frustrated in the event that the detection rate is too low in the subsequent intervention. However, in a BCI_offline_ modus a major concern is that since the movement is not detected in real time, possible changes in reaction time to the cue caused by factors such as attention shifts or fatigue will lead to a decreased accuracy in the timing between the peripherally generated afferent volley and the activation of the brain.

An important prerequisite in the associative BCI intervention we first introduced in 2012 in healthy participants (Mrachacz-Kersting et al., 2012) and later applied in a group of chronic stroke patients where it led to significant functional improvements (Mrachacz-Kersting et al., 2016), is thus that the PN of the MRCP is reliable across single trials. Typically, within a session, a training data set of 30–50 trials of attempted movements is performed and the extracted time of PN used in the subsequent intervention. The intervention is comprised of 30–50 pairings of an artificially generated afferent volley timed to arrive at PN. This timing is imperative as neither early nor late arrival results in plasticity induction (Mrachacz-Kersting et al., 2012). The average PN time in the initial training set was similar to what we have reported previously and did not differ significantly for the BCI_offline_ and BCI_online_ sessions (Mrachacz-Kersting et al., 2012, 2017c). Since participants did not alter their reaction time to the visual cue within the BCI_offline_ intervention set (the PN time was similar to the initial 30 trials), we may assume that indeed 30 pairs with the appropriate time were applied. However, for the BCI_online_ session, the TPR was only 71 ± 3% indicating that for almost 30% of the actual movements, no artificial volley was generated. In a self-paced BCI that follows the same principles of associativity the TPRs attained similar values of 67.15 ± 7.87% (Niazi et al., 2012) and 73.0 ± 10.3% (Xu et al., 2014).

In the previous self-paced associative BCI, participants were required to continue performing the task until at least 50 successful attempts were detected (Niazi et al., 2012; Xu et al., 2014). This number of pairings was based on previous studies of PAS targeting a hand muscle (Kujirai et al., 2006). As a result, the duration of the intervention session was between 8.9 and 22.1 min. In the current study, irrespective of the number of true detections, only 30 trials were completed with a total duration of approximately 5 min. In a BCI designed for neurorehabilitation of stroke patients it is imperative that each BCI session does not last longer than approximately 30 min. This includes all aspects such as preparation time, training and the intervention itself. This has several reasons, on the one hand, at least in Denmark, any therapy session for stroke patients takes maximally 30 min and maintaining the BCI session within this time frame will allow it to be scheduled alongside the classical therapy sessions. On the other hand, stroke patients fatigue at a faster rate compared to healthy controls with 30 min being the maximum time they are able to concentrate prior to necessitating a rest period.

### Changes in the Output Properties of the Motor Cortex Following the Associative BCI_offline_ and BCI_online_ Interventions

In the current study, participants were exposed to a significantly reduced number of pairings of the MRCP and the afferent inflow in the BCI_online_ intervention, compared to previous studies and the BCI_offline_ intervention. However significant plasticity of the corticospinal tract to the TA muscle occurred. It is currently not established how many pairs of peripheral and central inputs are required for such changes to be induced. In previous studies both 50 pairings (Mrachacz-Kersting et al., 2012, 2016) and 30 pairings (Mrachacz-Kersting et al., 2017c) have resulted in significant changes. In the original PAS studies (see review by Suppa et al., 2017), 90 pairs were applied when targeting hand muscles (Stefan, 2000), and this could be further reduced to 50 when the muscle was pre-contracted (Kujirai et al., 2006). As a minimum, 360 pairs were required when targeting the lower limb muscle TA (Mrachacz-Kersting et al., 2007) and 200 for soleus (Kumpulainen et al., 2012, 2015). At least for PAS, other factors such as attention to the task, fatigue and history of muscle contraction have been shown to contribute to the changes in the excitability of the cortical projections to the target muscle (Suppa et al., 2017). Thus, any attention away from the main task as well as fatigue will lead to a decrease in the amount of plasticity induced (Stefan et al., 2004), while prior muscle activation will lead to an increase (Kujirai et al., 2006). Since the duration and the number of trials performed were exactly the same for the BCI_online_ and BCI_offline_ intervention, it is likely that participants were able to attend to the task without experiencing attentional shifts or fatigue.

During the BCI_online_ intervention, a movement was falsely detected at a rate of 1.2 ± 0.9 min^-1^. Thus, on average six ES were not timed to the PN of the MRCP. Previously, afferent inflow that arrived either too early or too late resulted in no significant plasticity induction (Mrachacz-Kersting et al., 2012), while an ES timed randomly in relation to PN led to decreases of the excitability of the cortical projections to the TA in some chronic stroke patients while triggering no changes on average across all patients (Mrachacz-Kersting et al., 2016). These results taken together imply that although our associative BCI intervention is modeled on PAS and associative LTP-like mechanisms, there are likely significant differences in the locus of effects (Suppa et al., 2017). Further studies are required to determine the exact sites of plasticity. Lastly, since participants performed the task in both the training and intervention sets, afferent inflow was generated naturally by the activation of the muscles, arriving at the motor cortex at the appropriate time. This afferent feedback is a combination of muscle, joint, and skin receptor activation. It may be speculated that in the event that the artificially generated afferent volley occurs at the wrong time in relation to the MRCP, it is simply filtered out by the nervous system. This is supported by our original findings that afferent feedback timed either too early or too late in relation to the PN of the MRCP leads to no plasticity induction. It is also substantiated by reports that the effects of afferent feedback to the brain and ongoing movement is modulated in a task dependent manner (Nielsen and Sinkjaer, 2002; Nielsen, 2004). Thus for example, during an active dorsiflexion movement, afferent information from antagonistic muscles is suppressed by disynaptic reciprocal inhibition (Crone and Nielsen, 1994; Geertsen et al., 2011). Indeed, afferent feedback from the activation of ankle plantarflexors of one leg will depress the activation of the homonymous muscle of the other leg through a short latency interlimb pathway (Stubbs and Mrachacz-Kersting, 2009) that includes the same interneuron responsible for disynaptic reciprocal inhibition (Mrachacz-Kersting et al., 2017a).

## Conclusion

Here, we compared the effectiveness of an associative BCI_online_ and BCI_offline_ intervention in inducing plasticity of the cortical projections to the TA. Regardless of whether the PN of the MRCP was determined offline from a training data set or detected online, similar changes in the excitability of the cortical projections to the TA were induced. These results provide further strong support for the associative nature of the interventions but also suggest that they likely differ to the PAS protocol they were modeled on in the exact sites of plasticity. Further studies are required to assess whether the associative BCI_online_ and BCI_offline_ interventions have similar effects to PAS on the motor cortical network.

## Author Contributions

NM-K conceptualized and designed the study. NM-K and SA collected the data partly with a student group, analyzed the data, completed the statistical analysis and drafted the manuscript. All authors approved the final version.

## Conflict of Interest Statement

The authors declare that the research was conducted in the absence of any commercial or financial relationships that could be construed as a potential conflict of interest.

## Footnotes

1 seniam.org
